# Lymphopenia predicts 30-day morbidity and mortality following spinal metastasis surgery

**DOI:** 10.1016/j.xnsj.2021.100062

**Published:** 2021-04-15

**Authors:** Pedro Reggiani Anzuatégui, Glauco José Pauka Mello, Ana Valéria Brunetti Rigolino

**Affiliations:** Orthopedic Oncology Service, Hospital Erasto Gaertner, Rua Ovande do Amaral, 201, 81520-060 Curitiba, Paraná, Brazil

**Keywords:** Lymphocyte, Spine, Surgery, Metastasis, Survival, Complications, Mortality, Morbidity

## Abstract

**Background:**

Therapeutic decision-making regarding surgical treatment of spinal metastasis is supported by clinical characteristics that are potentially predictive of postoperative events. The predictive power of total lymphocyte count (TLC) in peripheral blood has not been elucidated for this type of surgery. Therefore, the aim of this study was to assess the capacity of TLC to predict 30-day morbidity and mortality following surgery for spinal metastases.

**Methods:**

This is a level III prognostic study, which consists of a retrospective review of records from a cancer referral hospital. Consecutive patients who underwent open surgery for spinal metastatic disease were studied. Outcomes of interest were 30-day post-op mortality and complications. The patients were divided into three groups based on preoperative TLC: low, moderate, and high risk for surgery, according to a discriminatory power analysis. The predictive power of TLC was compared to that of other known predictors, i.e., older age, tumor aggressiveness, and presence of comorbidities. Odds ratios (ORs) and 95% confidence intervals were calculated using bivariate and multivariate analyses.

**Results:**

In total, 205 patients underwent surgery. Thirty-day mortality and occurrence of complications were 17% and 31%, respectively. The discriminatory power of TLC was 71% and 68% for 30-day survival and complications, respectively. In multivariate analysis, the strongest relationship between predictors and postoperative morbidity and mortality concerned TLC < 800 cells/µL, which was associated with decreased likelihood of 30-day survival (OR 3.17) and increased likelihood of complications (OR 3.93). Incidence of 30-day mortality and complications by risk group was, respectively: 4% and 13% for low risk (TLC > 1857 cells/µL); 22% and 34% for moderate risk (TLC 800-1857 cells/µL); and 35% and 56% for high risk (TLC < 800 cells/µL).

**Conclusions:**

TLC is a strong predictor of 30-day morbidity and mortality following spinal metastasis surgery. It may be useful for improving patient care and planning personalized treatments.

## Introduction

Although good outcomes have been abundantly reported for spinal metastasis surgery, estimating surgical risk is often difficult because of the heterogeneous characteristics of patients, who are typically weakened. The therapeutic choice for a conservative or a surgical treatment is complex because of the high incidence of postoperative complications, which often reduce the limited survival of such patients [1]. Thus, several studies have assessed predictive factors of surgical outcomes in spinal metastasis. Traditional predictors concern clinical history and imaging test results, such as tumor aggressiveness, presence of visceral metastases, and number of bone metastases [2].

More recently, laboratory biomarkers have been shown to be predictive of outcomes in several medical conditions, especially cancer [3]. Total lymphocyte count (TLC) can be considered an immune-related biomarker because of its inclusion in prognostic scores such as the prognostic nutritional index (PNI), the neutrophil-to-lymphocyte ratio (NLR), and the platelet-to-lymphocyte ratio (PLR) [4]. TLC is a readily available indicator and has been found to be a significant independent predictor of poor outcomes in multiple conditions [5], [6], [7], [8], [9], [10].

Lymphopenia has been shown to be part of the natural history of cancer due to the association between malnutrition, myelosuppression, and lymphocyte recruitment in infectious and inflammatory processes [11]. However, the predictive power of TLC in spinal metastasis surgery has not been completely elucidated, especially in terms of ability to predict short-term postoperative events. Thus, this study aimed to investigate the predictive power of TLC regarding morbidity and mortality within 30 days of spinal metastasis surgery.

## Materials and methods

### Data source and inclusion criteria

We conducted a retrospective analysis of a cohort of consecutive patients surgically treated for spinal metastasis between January 1, 2002 and December 31, 2015. The study was approved by the ethics committee of our institution, and informed consent was waived because of its retrospective nature.

Inclusion criteria were: a) patients who underwent open surgery; and b) pathology test results confirming the diagnosis of malignant metastatic spinal tumor. Exclusion criteria were: a) percutaneous surgical procedures; b) primary surgery or revision surgery in another institution; c) incomplete medical record data; and d) loss to follow-up.

The selected cases were manually reviewed to obtain demographic data, including age at the time of presentation, sex, postoperative survival and complications, significant medical comorbidities using combined Charlson and Elixhauser indices [12], primary tumor diagnosis, type of approach, and preoperative TLC in peripheral blood. Thirty-day postoperative survival and development of major complications within 30 days of surgery were chosen as the outcomes of interest. The complications were characterized and classified using the method described by Rampersaud et al. [13].

### Lymphocyte count as an outcome predictor

In the present study, preoperative TLC was defined as the absolute lymphocyte count plus the enlarged atypical lymphocytes, such as reactive lymphocytes or lymphoblasts. TLC was considered a potential predictor of the outcomes of interest. Some known major predictors of poor postoperative outcomes were used for comparison purposes: age ≥ 70 years, presence of ≥ 1 significant comorbidity, and primary tumor not graded as slow-growing, according to Tomita et al. [14].

The patients were divided into three groups based on their preoperative TLC: low, moderate, and high risk for surgery. Youden's index for 30-day survival provided an optimal TLC level of 1858 cells/µL (sensitivity of 94% and specificity of 43%), which defined the low-risk group. TLC < 800 cells/µL was arbitrarily chosen for the high-risk group as it indicates severe malnutrition [15]. Consequently, the moderate-risk group was established when the TLC level was between 800 and 1857 cells/µL.

### Statistical analysis

Continuous variables were dichotomized and treated as categorical variables. Fisher's exact test and chi-square test were used for risk assessment. Discriminatory power was assessed using a receiver operating characteristic (ROC) curve. Youden's index was used to define optimal values for sensitivity and specificity. Kaplan–Meier method and log-rank test were used to elaborate and compare survival curves. By comparing the frequency of occurrence of outcomes in individuals exposed and not exposed to potential predictors and then performing a multivariate analysis, we were able to identify independent predictors, which were quantified by odds ratio (OR). Logistic regression models were applied to the groups of variables, provided that p < 0.05 in bivariate analysis. A 95% confidence interval was used in all analyses. R (R Foundation for Statistical Computing, Vienna, Austria), version 3.3.1 [16], and MedCalc (MedCalc Software, Ostend, Belgium), version 19.2.1 [17], were used for statistical tests.

## Results

### Patient characteristics, surgical outcomes, and ROC curve analysis

A total of 306 patients underwent surgery, of which 78 were excluded because there was no anatomopathological confirmation of metastatic disease. This occurred because it was not routine, for some years, the collection of intraoperative confirmatory tests in clearly metastatic patients. Of the 228 remaining patients, 18 were excluded due to incomplete data in medical records (in general, the date of death was missing), four were excluded due to loss of follow-up and one was excluded because it was a revision surgery from another service. Therefore, after the adoption of the inclusion and exclusion criteria, 205 patients were included in the study.

The demographic and surgical characteristics of the patients, as well as their outcomes, are presented in Table 1 according to TLC risk groups. Spinal decompression plus fixation was performed in all cases, and 95% of the patients underwent a posterior approach, aiming at the direct decompression of neural structures. Decompression of the anterior column from posterior was performed when necessary for thoracolumbar approach, and reconstruction of the anterior column was performed only for cervical approach.

**Table 1:** Surgical characteristics and outcomes of 205 patients surgically treated for spinal metastasis.

Variables	TLC < 800 cells/µL	TLC 800-1857 cells/µL	TLC > 1857 cells/µL	*p*-value
Number of patients	34	96	75	
Mean age, years (± SD)	63 (± 11)	59 (± 13)	57 (± 14)	0.07
Male	18 (53%)	52 (54%)	44 (59%)	0.52
Age ≥ 70 years	11 (32%)	22 (23%)	15 (20%)	0.19
Primary tumor not graded as slow-growing	13 (38%)	38 (40%)	30 (40%)	0.98
Presence of ≥ 1 significant comorbidity	13 (38%)	28 (29%)	24 (32%)	0.62
Diabetes	6 (18%)	11 (11%)	8 (11%)	0.56
Chronic lung disease	4 (12%)	10 (10%)	8 (11%)	0.98
Heart disease	3 (9%)	7 (7%)	7 (9%)	0.88
30-day outcomes				
Mortality	12 (35%)	21 (22%)	3 (4%)	< 0.0001
Total complications	19 (56%)	33 (34%)	10 (13%)	<0.0001
Infectious complications*	38%	20%	11%	0.006
Non-infectious complications*	10%	10%	3%	0.15

TLC, total lymphocyte count.

* Twelve cases were excluded of the analysis because infection could not be determined.

The most common type of approach was thoracolumbar (35%), followed by thoracic (34%), lumbar/lumbosacral (24%), cervical/cervicothoracic (5%), and multiple (2%). The frequency of histological subtypes of cancer was 24% prostate, 21% breast, 13% multiple myeloma, 10% unknown, 6% uterus, and 25% other.

Of 205 patients, 75 (37%) had TLC > 1857 cells/µL, 96 (47%) had TLC of 800–1857 cells/µL, and 34 (17%) had TLC < 800 cells/μL. Regarding known predictors, 48 (23%) patients were ≥ 70 years of age; 65 (32%) patients had ≥ 1 comorbidity; and 81 (40%) patients had primary tumors that were not graded as slow-growing.

**Fig. 1:**
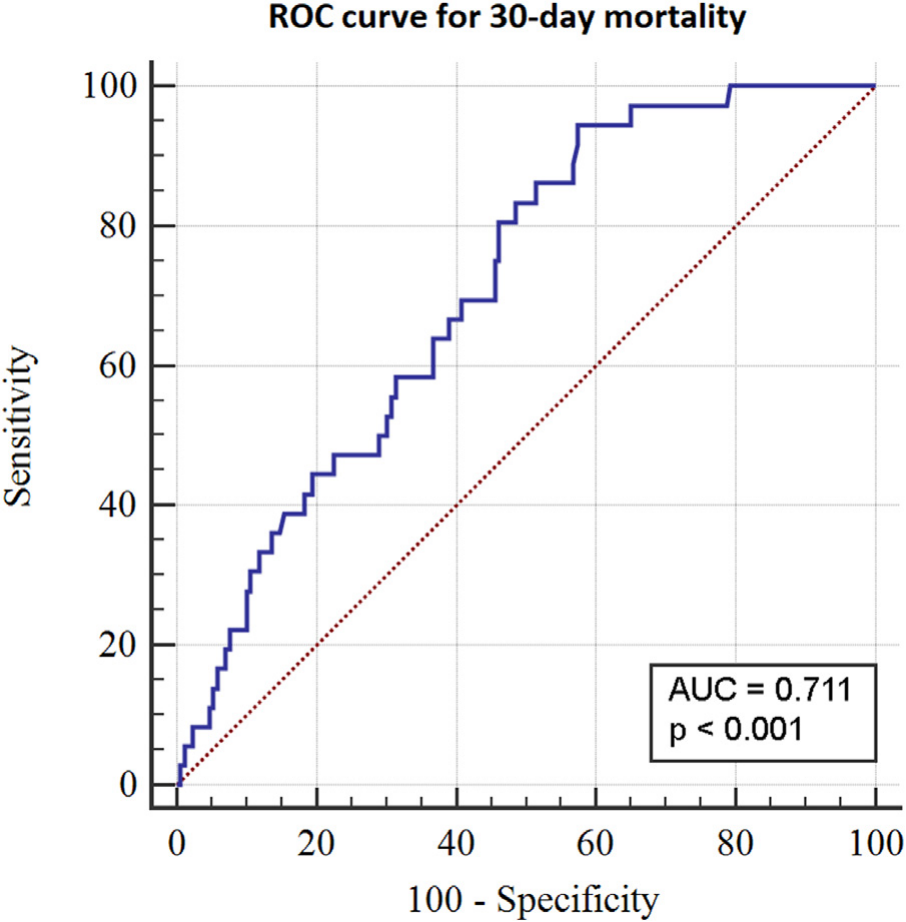
Receiver operating characteristic (ROC) curve showing the discriminatory power of total lymphocyte count (TLC) for 30-day mortality.

**Fig. 2:**
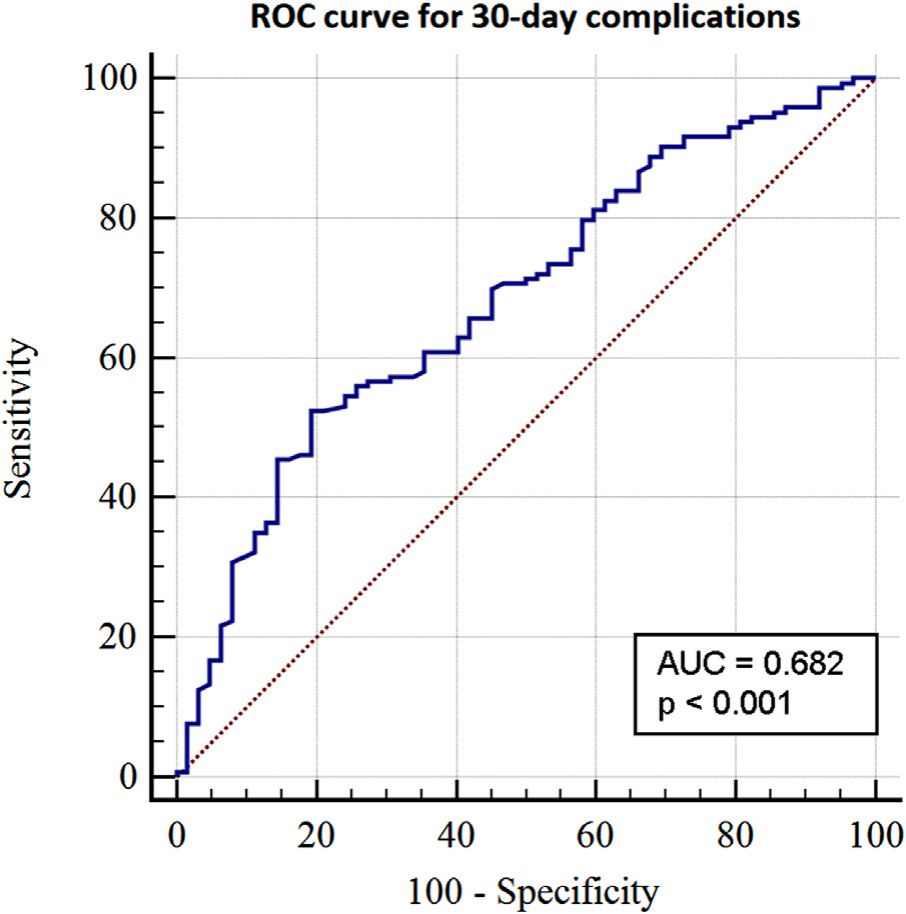
Receiver operating characteristic (ROC) curve showing the discriminatory power of total lymphocyte count (TLC) for postoperative complications.

Overall 30-day mortality was 17% (*n* = 36). The incidence of postoperative complications was 31% (*n* = 64), as detailed in Table 2. The discriminatory power of TLC was 71% (p < 0.001) and 68% (p < 0.001) for 30-day survival and occurrence of complications, respectively, as shown in Figs. 1 and 2.

**Table 2:** Incidence of complications following surgical treatment of spinal metastasis.

Complications	n (%)
Systemic	40 (19.5)
Pneumonia	14 (6.8)
Death of unknown cause	11 (5.4)
Gastrointestinal bleeding	4 (2.0)
Respiratory failure	3 (1.5)
Renal failure	2 (1.0)
Urosepsis	1 (0.5)
Other	4 (2.0)
Local	24 (11.7)
Wound infection	20 (9.8)
Wound dehiscence	2 (1.0)
Hematoma	1 (0.5)
Neurological impairment	1 (0.5)
Grade III	19 (9.3)
Grade IV	45 (21.9)
Total	64 (31.2)

Note: According to Rampersaud et al., a grade III complication requires significant treatment, i.e., increases hospital stay by more than 7 days and/or causes sequelae for more than 6 months. A grade IV complication is one that results in death.

### TLC and other predictors

In bivariate analysis, the following results were found for 30-day mortality: TLC < 800 cells/µL (OR 3.34; 95% CI 1.46–7.62; *p* < 0.01), TLC 800–1857 cells/µL (OR 1.75; 95% CI 0.85–3.64; *p* = 0.13), TLC > 1857 cells/µL (OR 0.12; 95% CI 0.04–0.41; *p* < 0.001), age ≥ 70 years (OR 2.94; 95% CI 1.37–6.32; *p* < 0.01), presence of comorbidities (OR 2.60; 95% CI 1.24-5.41; *p* = 0.01), and primary tumor not graded as slow-growing (OR 2.21; 95% CI 1.07–4.59; *p* = 0.03). Regarding the odds of developing postoperative complications, the results were as follows: TLC < 800 cells/µL (OR 3.77; 95% CI 1.76–8.06; *p* < 0.001), TLC 800-1857 cells/µL (OR 1.44; 95% CI 0.79–2.63; p = 0.22), TLC > 1857 cells/µL (OR 0.23; 95% CI 0.11–0.49; *p* = 0.0001), age ≥ 70 years (OR 3.13; 95% CI 1.60–3.14; *p* < 0.01), presence of comorbidities (OR 2.61; 95% CI 1.40–4.88; *p* < 0.01), primary tumor not graded as slow-growing (OR 2.48; 95% CI 1.35–4.56; *p* < 0.01).

Lymphopenia (TLC < 800 cells/µL), older age, presence of comorbidities, and tumor aggressiveness met the criteria for inclusion in the multivariate models for both 30-day survival and occurrence of complications, based on performance in bivariate analysis.

Tables 3 and 4 show the results of multivariate analysis for the outcomes of interest. In multivariate analysis, the strongest relationship between predictors and postoperative morbidity and mortality concerned TLC, which was associated with a decreased likelihood of 30-day survival and an increased likelihood of complications.

**Table 3:** Multivariate analysis of preoperative lymphopenia in peripheral blood as a potential predictor of 30-day mortality following spinal metastasis surgery.

Predictors	OR	95% CI	*p*-value
TLC < 800 cells/µL	3.17	1.32–7.65	0.01
Age ≥ 70 years	2.75	1.22–6.22	0.01
Presence of ≥ 1 significant comorbidity	2.30	1.06–5.01	0.03
Primary tumor not graded as slow-growing	2.39	1.10–5.21	0.03

Effect sizes are described as odds ratio (OR) with 95% confidence interval (CI). TLC, total lymphocyte count.

*Significant comorbidities as proposed by combined Charlson and Elixhauser indices.

**Table 4:** Multivariate analysis of preoperative lymphopenia in peripheral blood as a potential predictor of major complications following spinal metastasis surgery.

Predictors	OR	95% CI	*p*-value
TLC < 800 cells/µL	3.93	1.70–9.03	< 0.01
Age ≥ 70 years	3.18	1.51–6.71	< 0.01
Presence of ≥ 1 significant comorbidity	2.35	1.19–4.64	0.01
Primary tumor not graded as slow-growing	2.99	1.51–5.91	< 0.01

Effect sizes are described as odds ratio (OR) with 95% confidence interval (CI). TLC, total lymphocyte count.

*Significant comorbidities as proposed by combined Charlson and Elixhauser indices.

**Fig. 3:**
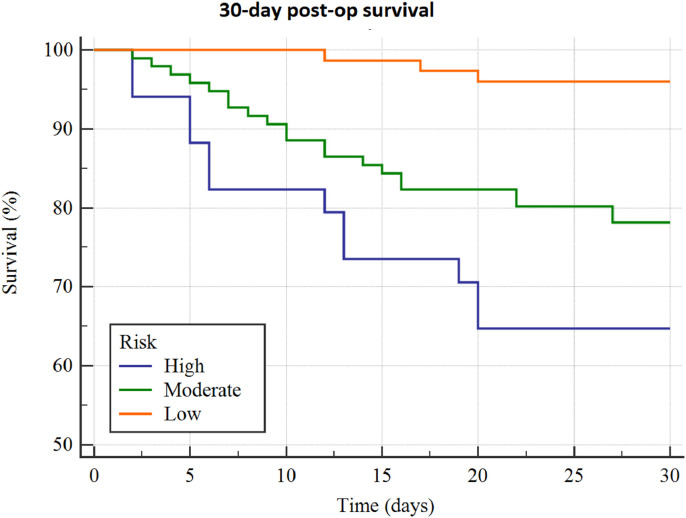
Kaplan–Meier curve showing the effect of preoperative total lymphocyte count < 800, 800-1857, and > 1857 cells/µL in peripheral blood on 30-day survival following spinal metastasis surgery.

**Fig. 4:**
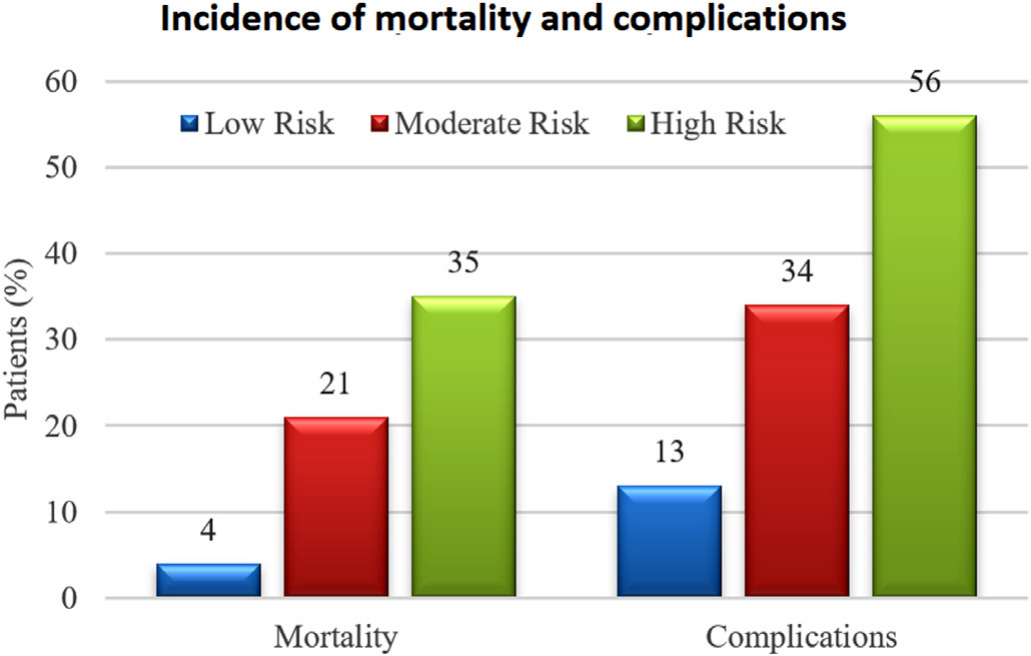
Thirty-day morbidity and mortality following surgical treatment for spinal metastasis according to preoperative total lymphocyte count in peripheral blood.

The influence of each TLC risk range on postoperative 30-day survival is shown in Fig. 3. The incidence of mortality and complications within 30 days of surgery by risk group was, respectively, 4% and 13% for low risk, 22% and 34% for moderate risk, and 35% and 56% for high risk (*p* < 0.0001, Table 1 and Fig. 4).

## Discussion

Lymphopenia has not yet been associated with poor early outcomes in spinal metastasis surgery. In this retrospective analysis of 205 consecutive patients surgically treated for spinal metastasis, we showed that preoperative TLC in peripheral blood strongly predicts morbidity and mortality within 30 days of surgery, being the most relevant factor among other known predictors.

Patients with spinal metastasis are usually weakened, but their clinical presentation varies widely [1]. TLC is a simple tool that can be used to estimate the patient's ability to recover from a major surgery, as it indicates how prepared the patient is to endure a postoperative intensive care unit (ICU) stay without developing pneumonia [5] or other life-threatening infectious or non-infectious complications [18].

Lymphopenia has been shown to be related to the natural history of cancer. Yakovlev et al. [11] found that mean TLC in kidney cancer reduced by 20% from the initial to the final stage of the disease. Several associated conditions may explain this phenomenon, including mineral depletion in malnutrition, cell recruitment in inflammatory or infectious processes, myelosuppression due to chemotherapy or radiotherapy, and induction of T-cell apoptosis [11].

Previous studies have shown that lymphopenia is associated with poor medium- and long-term survival in patients with cancer [6,19]. To the best of our knowledge, this is the first study to demonstrate the early predictive power of TLC in spinal metastasis surgery. We believe that unfavorable outcomes within 30 days of surgery are more related to complications and the patient's ability to recover than to tumor progression.

Because malnutrition is a common finding in orthopedic oncology and its role in unfavorable surgical outcomes is well known [18], preoperative screening with TLC measurement has been suggested. Zinc and other vitamins act on cell maturation, and vitamin deficiency may partially explain the presence of lymphopenia in severely malnourished patients [20].

TLC and serum albumin are traditional nutritional markers that can be used clinically, either alone or via PNI using the following equation: PNI = (10 x albumin g/dL) + (0.005 x TLC µg/dL). The clinical value of serum albumin alone has been widely studied, and its predictive power for the occurrence of orthopedic complications has been established at levels < 3.5 mg/dL [18,21]. No cut-off point has been determined for TLC regarding the prediction of clinical outcomes. In this study, we used 800 cells/µL as a cut-off point for the high-risk group (an indicator of severe malnutrition) [17] and 1857 cells/µL for the low-risk group (Youden's index). Previous studies have found clinical relevance at different levels (500–1900 cells/µL) [8,22].

A low TLC level before any medical intervention is a well-known predictor of mortality [5], [6], [7], [8], [9], [10]. Studies have shown an increased risk of death in ICU-acquired pneumonia (hazard ratio, HR 1.41; TLC < 595 cells/µL) [5], cardiac surgery (OR 1.01 for each reduction of 10 cells/µL in TLC) [7], intestinal surgery (HR 9.09; no cut-off point was defined) [10], gastric cancer surgery (HR 1.97; TLC < 1462 cells/µL) [6], chemotherapy for melanoma (unspecified risk; TLC < 1500 cells/µL) [23], clinical treatment of lung cancer (HR 1.81; TLC < 1900 cells/µL) [8], surgery for femoral neck fractures (OR 1.45 for each reduction of 100 cells/µL in TLC) [9], and surgery for appendicular pathological fractures (unspecified risk; TLC < 600 cells/µL) [19].

Only one study other than ours has assessed the predictive power of TLC for postoperative survival of patients with spinal metastasis. However, the results are not comparable because of the use of different methods. Of the 17 variables considered potentially predictive by Karhade et al. [24], TLC ranked fourth among the most important predictors of 90-day mortality, following hypoalbuminemia, tumor aggressiveness, and low oncology performance score. The predictive power of low TCL was equivalent to 40% of that associated with hypoalbuminemia, the most relevant predictor. Karhade et al. [24] also observed that, although relevant, the predictive power of TLC was lower for 1-year mortality. This finding reinforces our hypothesis that the predictive power of lymphopenia is related to the patient's early ability to recover from surgery.

Our results suggest that short-term mortality following spinal metastasis surgery is strongly related to preoperative TLC in peripheral blood, as only 4% of patients in the low-risk group (TLC > 1857 cells/µL) died within 30 days of the procedure compared to 35% in the high-risk group (TLC < 800 cells/µL). These numbers represent a three-fold higher risk of short-term death in patients with severe lymphopenia. Future studies may support or contradict these findings, as there is no comparative data available at the moment.

To the best of our knowledge, this is the first study to assess the discriminatory power of lymphopenia to estimate postoperative survival of patients with spinal metastasis. We found an accuracy of 71% for 30-day mortality, and other studies reported similar numbers: 83% for 30-day mortality in cardiac surgery [7], 74% for 90-day mortality in ICU-acquired pneumonia [5], and 61% for overall survival in gastric cancer surgery [6].

There is a lack of studies assessing postoperative complications. Additionally, methodological differences and underreporting of negative results make comparisons more difficult. We could not find studies addressing the influence of lymphopenia on the occurrence of complications following spinal metastasis surgery.

In cardiac surgery, preoperative TLC < 1485 cells/µL has proved to be useful for predicting morbidity, as Agahdaii et al. [7] reported an impressive accuracy of 88%. Ninety-six percent of 26 patients with TLC < 1000 cells/µL before cardiac surgery had some degree of morbidity; conversely, this outcome was reported in only 8% of patients with TLC > 1500 cells/µL. Chiarelli et al. [10] investigated 21 potential predictors of major complications in intestinal surgery, and only two of them were found to be predictive of unfavorable outcomes: lymphopenia (OR 2.00) and rectal resection (OR 2.83).

In our study, only major complications were assessed. A low TLC level was associated with infectious complications, which suggests the importance of assessing the patient's immune status when spinal metastasis surgery is indicated. Our results show that TLC < 800 cells/µL is related to a substantial four-fold higher risk of postoperative complications. It is worth noting that Rampersaud grade IV complication (i.e., death) is included.

The ROC curve for accuracy of TLC in predicting postoperative complications showed a value of 68%, which is satisfactory considering that measuring lymphocytes in peripheral blood is quite simple. Further research is needed to support the significance of these results.

Several recent studies have demonstrated the importance of inflammatory biomarkers in cancer. Inflammation is assumed to play a role in carcinogenesis and tumor progression. The activity of the immune system leads to a change in leukocyte profile, which acts as a natural marker of systemic inflammation [3]. Indirectly, TLC can be considered a biomarker because it is included in widely studied prognostic scores that are related to cancer prognosis.

Proctor et al. [4] examined a large sample of 27031 patients and found that the following biomarkers were associated with cancer survival regardless of tumor histology: PNI, NLR, PLR, modified Glasgow prognostic score (mGPS), and prognostic index (PI). TLC is included in PNI, NLR, and PLR. The usefulness of PLR has been demonstrated for predicting survival within 6 and 12 months, treatment failure, and readmissions in patients with spinal metastasis [25]. Also, in the study conducted by Karhade et al. [24], a poor prognosis indicated by TLC seems to be related to postoperative complications (i.e., short-term outcomes), while a poor prognosis indicated by other inflammatory biomarkers seems to be related to tumor progression (i.e., medium- and long-term outcomes).

Several prognostic tools have been developed for accurate estimation of postoperative survival in spinal metastasis surgery [26]. Preoperative clinical characteristics that are predictive of short-term outcomes (i.e., within 30 days) may differ from those of medium-term (i.e., within 90 days) and long-term outcomes (i.e., within 1 year). Thus, recent studies have included different laboratory biomarkers such as albumin [27,28], leukocytes [29], NLR [24], PLR [24], and TLC [24,12] in their predictive models, resulting in more accurate findings.

In a previous study conducted by our research group [12], we developed a prognostic score with TLC as a predictor for use in therapeutic decision-making regarding spinal metastasis surgery. The tool is quite simple and achieved an accuracy > 70% for 90-day morbidity and mortality. The process of validation of that study is currently in progress. Karhade et al. [24], in turn, developed a complex prognostic tool based on machine learning that is available as a web application. By combining traditional clinical characteristics and modern biomarkers (including TLC), Karhade's prognostic tool achieved an accuracy of 83% and 89% for 90-day and 360-day mortality following surgery, respectively.

Our study has some limitations. Because of its retrospective nature, selection, measurement, and susceptibility bias may have occurred. The results may not be generalized because the study was conducted at a single institution in Brazil, which means potential for homogeneous clinical decision-making and surgical practice. The typically heterogeneous characteristics of patients with spinal metastasis may lead to different clinical behaviors that may compromise a comparison between individuals. The major strength of our study is its large cohort of patients.

In conclusion, identifying TLC as a predictor of morbidity and mortality following spinal metastasis surgery may be useful for improving patient care and planning personalized treatments. It might also lead to the identification of therapeutic drugs that could promote an immune response in selected cases.

## Financial disclosure

The authors have no financial relationships relevant to this article to disclose.

## Declaration of Competing Interest

The authors have no conflicts of interest to disclose.
